# Future prospects for image-derived input function in molecular imaging quantification with the 7 T MR-BrainPET insert

**DOI:** 10.3389/fnins.2025.1725728

**Published:** 2026-01-05

**Authors:** Cláudia Régio Brambilla, Julia Hilgers, Usman Khalid, Ninea Anasovi, Tornike Kikacheishvili, Jörg Mauler, Chang-Hoon Choi, Jürgen J. Scheins, N. Jon Shah, Christoph W. Lerche

**Affiliations:** 1 Medical Imaging Physics, Institute of Neuroscience and Medicine 4, INM-4, Forschungszentrum Jülich GmbH, Jülich, Germany; 2 Engineering, Agricultural University of Georgia, Tbilisi, Georgia; 3 Informatics and Engineering School, Georgian American University, Tbilisi, Georgia; 4 Department of Neurology, RWTH Aachen University, Aachen, Germany; 5 Institute of Neuroscience and Medicine 11, INM-11, Forschungszentrum Jülich GmbH, Jülich, Germany; 6 JARA-BRAIN, Aachen, Germany

**Keywords:** UHF 7 T MR, BrainPET 7 T insert, molecular imaging, neuroimaging, image-derived input function, PET quantification

## Abstract

Positron emission tomography (PET) and magnetic resonance imaging (MRI) offer complementary information about the human brain in health and disease. The simultaneous 7 T MR-BrainPET insert enables molecular imaging quantification beyond the current limits. Here we present the current status of the field highlighting PET/MR synergies for the image-derived input function (IDIF). We also discuss promising applications that will benefit from these advancements, as well the challenges to be addressed in the near future.

## 1 Introduction

*In vivo* neuroimaging techniques have progressed significantly in recent decades, advancing understanding of neural processes in health and disease (Jones et al., 2012; Fink and Schneider, 2007). A major step toward simultaneous multimodal imaging was achieved with the combination of PET and MRI (Schlemmer et al., 2008), and has been continuously developed since (Herzog and Lerche, 2016). While MRI enables precise access to neuroanatomy, flow dynamics and several other functional mappings, its sensitivity is insufficient for molecular imaging, which studies the biodistribution of molecular targets and metabolic processes *in vivo*. Compared to MRI, PET has a million-fold higher detection sensitivity and is, therefore, the most important, non-invasive molecular imaging modality. The pico-molar detection sensitivity of PET enables imaging of neurotransmitter/receptor concentrations, and potentially allows the evaluation of endogenous neurotransmitter fluctuations—stimulated by pharmacologic or cognitive challenges (Kegeles and Mann, 1997). Most brain-PET scans are performed with whole-body scanners, which requires trade-offs in the image performance. Therefore, several brain-dedicated scanners have been developed (Caldeira et al., 2019; Catana, 2019; Li et al., 2024).

**Figure 1:**
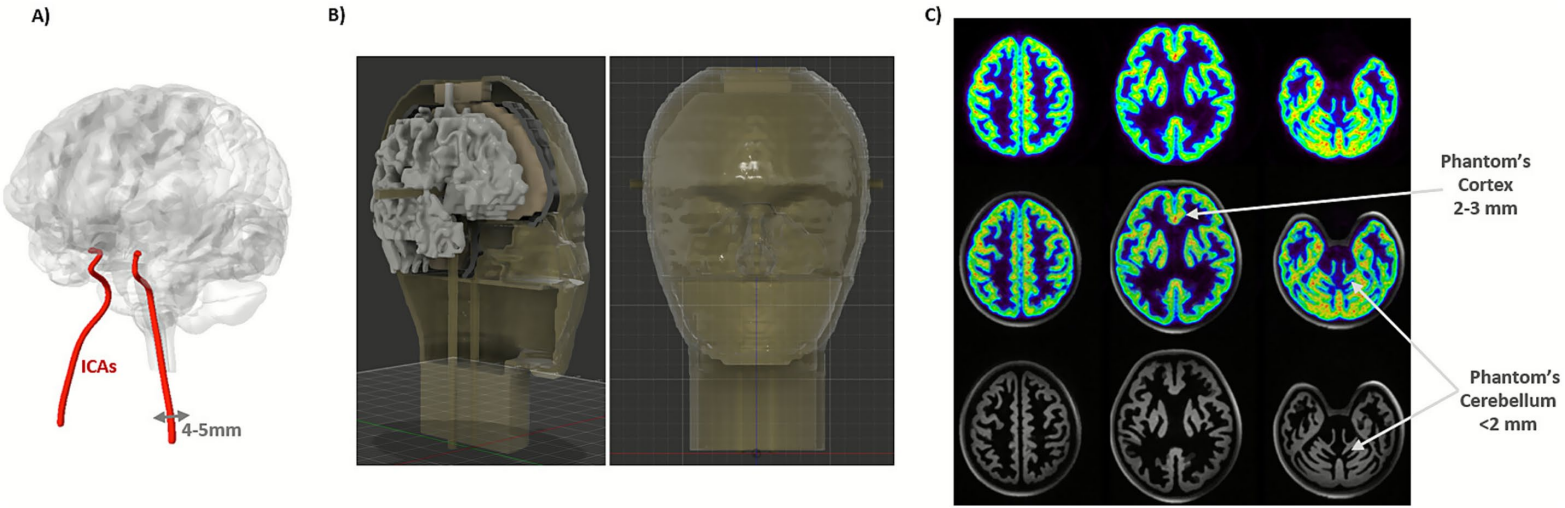
**(A)** Schematic visualization of the brain and ICAs. **(B)** Designed anthropomorphic head phantom with ICAs (updated from phantom shown in **C**). **(C)** Head phantom (Iida et al., 2013) acquisition in the 7 T MR-BrainPET showing the cortex and cerebellar structures in contrast with the human head from Figure 2A bottom (upper: BrainPET 7 T image, middle: PET/MR fused image and bottom: 7 T MR MP2RAGE T_1_-weighted image).

Brain-dedicated PET scanners are adapted to brain geometry, with reduced ring diameter (solid angle coverage) and thicker scintillation crystals leading to a better sensitivity and smaller scintillation crystals leading to a better spatial resolution. High detection sensitivity and time resolution are needed for a good image signal-to-noise ratio (SNR), quantification precision, and allow short acquisition intervals in the case of dynamic studies. The high spatial resolution of brain-PET scanners enables reliable region delineation and minimizes the partial volume effect (PVE) for the human cortex. As the cortex is highly folded, the spatial resolution should, ideally, be homogeneous over the entire field-of-view (FOV). Another important advantage of improved image-derived input function (IDIF) performance is it avoids the need for arterial cannulation, as the internal carotid arteries (ICAs) can be resolved in the PET image and used as input for the full quantification with kinetic modeling (Figure 1; Volpi et al., 2024). In addition, temporal resolution is very important, since for IDIF the whole-blood curves can be sampled with shorter image frames to better estimate its peak (<2–5 s; Kang et al., 2025; Volpi et al., 2023).

Despite the advantages of brain-dedicated PET scanners, a disadvantage is the increased parallax error whenever the scintillation detectors are not prepared to estimate the depth of interaction (DOI) of the *γ*-photons (Lerche et al., 2018). Additionally, care has to be taken to implement adequate data corrections to minimize motion effects, *γ*-photon attenuation, and scattering to assure high quantification fidelity.

## 2 Instrumentation innovations in MR and brain PET imaging

Compared to conventional field strengths (1.5 and 3 T), 7 T MRI offers increased SNR and specificity (Feinberg et al., 2023). This enables imaging with both higher spatial and temporal resolutions, allowing for better characterization of subtle and prominent anatomical features and microvascular structures. The benefits of 7 T (Springer et al., 2016; De Cocker et al., 2018; Feng et al., 2022; Koning et al., 2015; Zhu et al., 2016) have been demonstrated in various applications, with the focus here on the brain and the ICA imaging.

Accessing superior visualization of tiny intracranial branches, e.g., vessel walls, enables more specific assessment of properties, such as wall thickening and other subtle vascular characteristics that are hardly identifiable at lower fields, leading to more accurate images. To provide a comprehensive characterization of the vessel wall, modified turbo spin-echo (van der Kolk et al., 2013; Blankena et al., 2016; Zhu et al., 2018; Kleinloog et al., 2014) and time-of-flight (TOF; Park et al., 2018; Cosottini et al., 2024; von Morze et al., 2007), contrast-enhanced (Osuafor et al., 2022; Harteveld et al., 2015) and phase-contrast (Kang et al., 2016). MR angiography techniques are frequently employed.

One possible method for visualizing the ICA without using a contrast agent is TOF MR angiography (TOF-MRA), which provides precise localization information on the arterial inflow. TOF-MRA at 7 T has been shown to improve contrast-to-noise ratio (CNR) and thus visualization of cerebral arteries compared to 3 T (von Morze et al., 2007; Dimitrakopoulou-Strauss et al., 2021). This offers the possibility for higher resolution TOF-MRA with more precise delineation of the ICA lumen, enabling more accurate correction of the PVE and segmentation of the PET images to improve the IDIF estimation in MR-driven techniques.

A common challenge in cross-calibrating IDIF data to bolus injections and blood sampling is correcting for dispersion due to varying flow velocities across the vessel cross-section and course (Dimitrakopoulou-Strauss et al., 2021).

The advanced speed-accelerated 4D flow sequence (Schmitter et al., 2020) provides valuable haemodynamic information within the ICAs that improves our understanding of cerebrovascular disease progression. However, (B_1_^+^) inhomogeneity can be a challenge, but using a robust, large-dynamic-range parallel-transmit mapping technique to enable B_1_-shimming enhanced ICA image quality (de Buck et al., 2024). Furthermore, the introduction of artificial intelligence (AI)-driven models has advanced vascular imaging data analysis, enabling precise vessel identification and localization (Banerjee et al., 2024), and supporting an automated workflow that reduces reliance on inconsistent MRA data.

Despite the advantages afforded by 7 T MRI, the higher field strength increases the susceptibility to artifacts caused by arterial and cerebrospinal fluid (CSF) pulsation (Seginer et al., 2024), which reduces the accurate delineation of the vessel volume due to signal loss, ghosting artifacts and blurring. To minimize these artifacts, cardiac gating and motion compensation techniques can be used (Kuo et al., 2019).

Several projects are developing simultaneous PET/MR imaging at 7 T, aiming for full MR compatibility, significantly increased spatial resolution (1-2 mm), and increased sensitivity compared to standard clinical PET scanners (Allen et al., 2024; Won et al., 2021; Lerche et al., 2023). To achieve high spatial resolution across the entire PET FOV, the aforementioned scanners use DOI-capable scintillation detectors, which reduce parallax errors.

The cortex and ICA are of interest in neuroscientific applications, with average thicknesses or internal diameters of 2-5 mm and 4-5 mm, respectively (Baz et al., 2021). A noticeable PVE occurs in these structures when they are smaller than twice the spatial resolution of the system (Hoffman et al., 1979), requiring a spatial resolution around 1-2 mm to minimize this. On the other hand, increased detection sensitivity enhances image SNR, reducing statistical errors in the PET quantitative measurements, e.g., the rate constants required for compartment kinetic modeling (Dahlbom et al., 2005). Additionally, higher SNR enables shorter image frames for denser sampling of the radiotracer’s vascular phase, crucial for IDIF, which lasts ~90 s. Currently available PET scanners only allow very coarse sampling, resulting in an inaccurate determination of the bolus activity concentration, leading to error propagation during quantification. Reducing the sampling interval (currently ~15 s), while simultaneously increasing the SNR, would significantly reduce these errors. The PET image SNR can also be improved by taking TOF information into account during image reconstruction, and coincidence resolving time (CRT) has improved considerably to 200 ps in recent years. The BrainPET 7 T insert developed at the Forschungszentrum Jülich (FZJ) achieves a spatial resolution of 1.6 mm, a sensitivity of 11%, and a CRT of 620 ± 6 ps (Niekämper et al., 2025). This corresponds to a 2–3 times better resolution and is more than double the sensitivity of the BrainPET 3 T insert (Caldeira et al., 2019). At the same time, the BrainPET 7 T achieves a detection rate for the noise equivalent counts that is ~10 times higher than that of the BrainPET 3 T. Similar values are expected with the Human Dynamic NeuroChemical Connectome (HDNCC) scanner (Allen et al., 2024). However, due to the use of DOI-capable scintillation detectors, the two 7 T inserts under development have poorer CRT.

### 2.1 Brain PET in image quantification

For over 15 years, scientists at FZJ have advanced PET/MR brain imaging using one of only four prototype hybrid commercial 3 T MR-BrainPET inserts available worldwide (Herzog et al., 2011). Multiple tracers, including [^18^F]FDG, [^18^F]FET, [^15^O]H_2_O, have been employed alongside diverse MR sequences in clinical studies (Caldeira et al., 2019; Rajkumar et al., 2021; Mauler et al., 2024; Zhang et al., 2014; Mauler et al., 2020; Régio Brambilla et al., 2022).

In the context of IDIF, studies have applied semi-automatic ICA segmentation, PVC, new reconstruction methods, and dual simultaneous PET/MR acquisitions (Caldeira et al., 2015; da Silva et al., 2015; Anasovi et al., 2024). Most recently, AI has been applied in our studies for segmentation (Hilgers et al., 2025). In a pilot study with retrospective [^15^O]H_2_O data (Zhang et al., 2014), we achieved a volume of distribution (V_T_) agreement of R^2^ = 0.95 and 0.98 in gray matter (GM) and white matter (WM), when compared to V_T_ using arterial input function (AIF). However, microparameters showed quantification biases of 6.5–10.5%.

**Figure 2:**
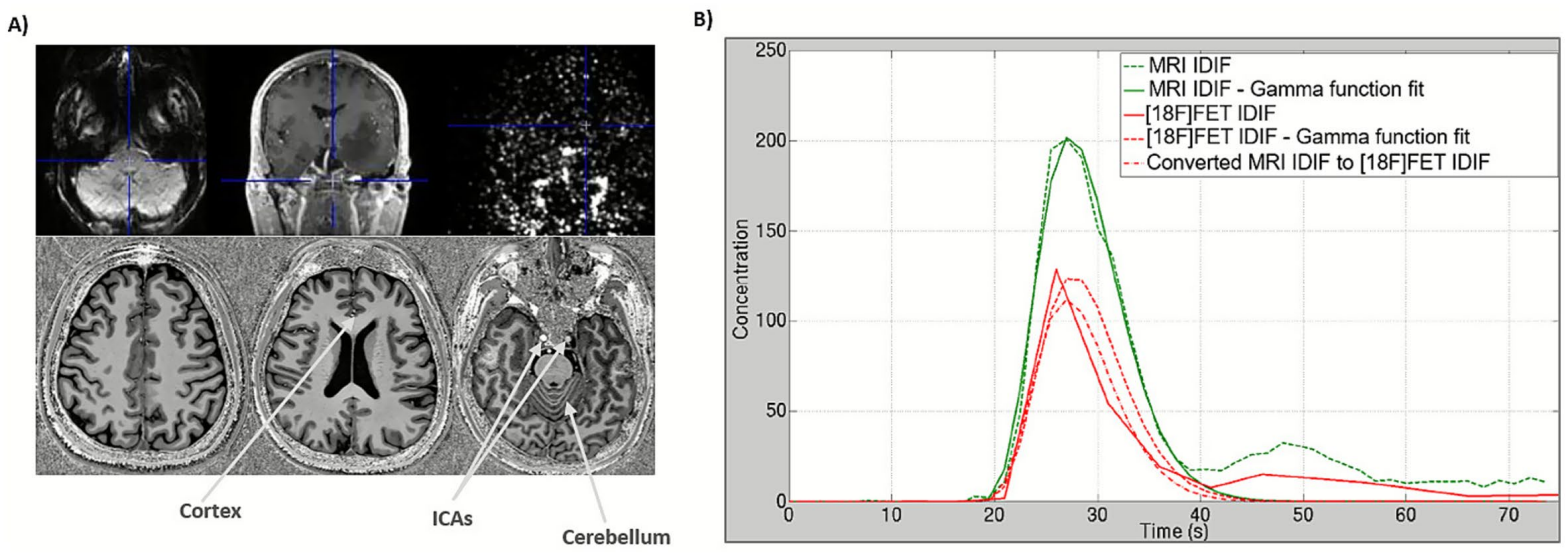
**(A)** Upper row from left to right: transaxial contrast-enhanced EPIK image, coronal MPRAGE T_1_-weighted structural image at 3 T MR showing the structure of the ICAs with contrast, and coronal [^18^F]FET 4th frame with 5 s. Bottom: transaxial MP2RAGE T_1_-weighted structural image at 7 T MR clearly showing the structure of the ICAs, cortex, and cerebellum without contrast agents. **(B)** Plots with IDIF curves from each modality with Gamma function fits and conversion from MRI IDIF to PET IDIF (estimated with 3 T MR-BrainPET upper images in **A**). Figure adapted from Caldeira et al. (2015).

The feasibility of combined and individual IDIF derivation from both, simultaneously acquired PET and MR data has already been demonstrated by extracting IDIF curves from 3 T MR EPIK (Yun et al., 2013) images with Gd-DTPA contrast and BrainPET images with [^18^F]FET (Figure 2), as well as by evaluating the relationships between the fit parameters obtained with both modalities (Caldeira et al., 2015). The shape (two parameters) and amplitudes (one parameter) of both curves in Figure 2 display linear relationships with agreements of R^2^ = 0.86, R^2^ = 0.98 and R^2^ = 0.92, respectively. Using these relationships, a conversion between the MRI and PET IDIF curves can be achieved, and a considerable potentiation of the joint determination can be expected at higher field strength (7 T vs. 3 T) and the improved imaging performance of the BrainPET 7 T component (spatial resolution 1.6 mm vs. 2 to 6 mm and sensitivity of 11% vs. 7%; Kolb et al., 2012).

Methodologically, enhancing PET/MR performance will be a vital addition to current IDIF approaches. These approaches have demonstrated that ICAs-based IDIFs—augmented by standardized PVC, motion correction, and small sets of blood samples for residual corrections—can approach arterial-line performance for many radiotracers. In addition, PET quantification can be further improved by using MR data from 7 T for automatic carotid segmentation, PVC, and refined estimation of IDIF (Caldeira et al., 2015).

### 2.2 Vessel segmentation and partial volume effects

The segmentation task of small vessel diameters like ICAs in brain PET images is currently challenging because of their diameter, which may lie at the limit of the spatial resolution of most PET scanners (~3–5 mm), their tortuous shape, and pulsation. Therefore, small intracranial vessels have traditionally been segmented manually or semi-automatically, but this may change in the light of the new scanners. Methods such as k-means (Liptrot et al., 2004), soft-decision similar component analysis (Brankov et al., 2003), local mean analysis (Maroy et al., 2008), region-growing (Galovic et al., 2021) and others have already been validated (Sundar et al., 2019; Naganawa et al., 2005; Chavan et al., 2024) for a limited number of tracers.

The U-Net topology is a widely used convolutional neural network for biomedical image segmentation (Ronneberger et al., 2015), with the newer version, nnU-Net, demonstrating excellent accuracy with Dice Coefficients up to 0.97 when segmenting blood vessels (Zhu et al., 2022).

In our pilot studies for estimating the IDIF using images acquired from the 3 T MR-BrainPET insert, the U-Net model achieved a Dice coefficient of 0.82, while the nnU-Net model achieved a Dice coefficient of 0.64 compared to the manually segmented reference. The PVE, resulting from the small ICA structure and limited PET scanner resolution, leads to activity spill-out (signal loss in ICA) and spill-in from neighboring tissues, leading to systematic absolute quantification errors in the IDIF estimations compared to the AIF. Several methods have been implemented for PVC, reducing the need for blood samples or estimating a recovery coefficient based on the vessel volume and scanner resolution (Silvestri et al., 2022; de Salvi Souza et al., 2025). Options include arterialized or venous samples when equilibrium occurs during the acquisition (Galovic et al., 2021; van der Weijden et al., 2023).

Combining superior MR images with higher native contrast (TOF-MRA), without contrast agents, alongside improved PET performance, would enhance PET quantification overall. Higher-quality images would reduce or eliminate the need for extensive pre-processing, increase segmentation accuracy (e.g., minimize tissue fraction effects due to limited sampling), and ultimately speed up and streamline the research pipeline.

We believe that with this synergy, it could be possible to exceed a Dice score of 0.90 with the U-Net and nnU-Net architectures and, moreover, establish a robust IDIF estimation.

### 2.3 Reconstruction

Apart from hardware improvements and data corrections, image reconstruction algorithms significantly impact final image quality and quantification accuracy. In this regard, the BrainPET 7 T insert poses a challenge due to the number of LORs (5 × 10^9^), which is addressed by the multi-layer DOI detectors. Here, the PET Reconstruction Software Toolkit (PRESTO) is ideally suited to deal with the inherent trade-off between accuracy and numerical effort in iterative reconstruction by applying scanner-independent, adaptive projection data (Scheins et al., 2011) in combination with speed-optimized projectors (Scheins et al., 2015). As a further asset, PRESTO supports Maximum-aposteriori (MAP) reconstruction using prior knowledge. In future work, specific vessel delineation, as provided by MR, could be exploited to minimize PVE. In terms of data correction accuracy, PET requires the estimation of background from Compton scattering, which can easily exceed 30% of the total statistics in neuro applications. Meanwhile, Monte Carlo simulations become feasible (Scheins et al., 2021) in dynamic imaging, potentially replacing Single Scatter Simulation, which is known to have limited accuracy and some pitfalls (Ma et al., 2020).

### 2.4 Multi-tracer applications

Multi-tracer imaging represents a promising advancement for assessing multiple physiological processes in a single scan session. By leveraging high-resolution PET systems and optimized signal processing algorithms, this approach can separate overlapping tracer signals, reducing patient burden and scan time compared to traditional protocols. For instance, studies have demonstrated the feasibility of triple-tracer imaging using synthetic data from clinical datasets (Hu et al., 2025).

Emerging technologies, such as those incorporating prompt gamma detection using ML-EM reconstruction methods, further enable precise multi-tracer imaging for studying complex brain disorders (Pfaehler et al., 2024). However, in multi-tracer brain PET, the IDIF faces challenges, as the overlapping signals complicate the accurate extraction of individual TACs essential for kinetic modeling. That being said, advanced signal decomposition techniques can potentially isolate tracer-specific contributions from vascular regions. Recent updates on IDIF methods highlight their non-invasive potential for human brain PET studies, noting that while ICA-based IDIF is viable for single tracers like [^15^O]H_2_O and [^18^F]FDG, extensions to multi-tracer scenarios will face new challenges to account for PVE, metabolite corrections (Volpi et al., 2023) and to minimize mixing effects in vascular structures (Vestergaard et al., 2021).

### 2.5 Phantoms for validation of IDIF estimation

Despite advances in IDIF, robust validation remains a critical challenge, particularly due to PVE (Zanotti-Fregonara et al., 2009). Dynamic phantoms incorporating realistic arterial structures and flow simulation are essential for validation. However, although most commercial head phantoms are designed for static anatomical simulation, attenuation correction, and lack dynamic flow, some prototypes simulate large vessels or modular flow-tube phantoms to systematically study IDIF recovery (Driscoll et al., 2022). Nevertheless, no commercially available dynamic head phantom with ICAs currently exists. To address this gap, we have recently developed a CT-derived, 3D printed prototype based on an existing head phantom (Iida et al., 2013). The phantom contains different fillable compartments (GM and WM), with incorporated ICAs that allow dynamic flow simulation through the phantom during PET/MR acquisitions. Additionally, we designed a cylindrical geometric phantom with an ICA insert (Ramnarine et al., 1998) as a reliable platform for IDIF validation.

Looking ahead, further improvements are envisioned, especially with the 7 T MR-BrainPET insert. Advanced 3D printing and multi-material techniques now allow more complex, accurate geometries and realistic flow simulations, leading to more reliable IDIF-based quantification (Liaw and Guvendiren, 2017).

## 3 Remaining challenges

### 3.1 Motion

Head motion during PET imaging can lead to image blurring, artifacts, and inaccurate quantification of tracer uptake and distribution. This degrades spatial resolution, reduces signal intensity in high-uptake regions, e.g., the brain cortex, and introduces bias in kinetic modeling (Spangler-Bickell et al., 2022). Common effects include motion-induced bias in binding potential estimates and overall reduced image quality, which is particularly problematic in dynamic scans lasting usually 30–90 min, where involuntary shifts from discomfort or neurological symptoms are common (Montgomery et al., 2006). To mitigate this, various correction strategies have been developed (Spangler-Bickell et al., 2022; Iwao et al., 2022; Wang et al., 2024; Chiu et al., 2025; Chen et al., 2021; Zeng et al., 2023; Ullisch et al., 2012).

The advent of next-generation systems amplifies the necessity for robust motion correction to preserve the benefits of enhanced sensitivity and spatial resolution. This correction is indispensable, because these scanners are inherently more sensitive to head displacements (~2 mm and 1^0^), which can obliterate the improved contrast and quantitative accuracy promised by such technology. This perspective underscores the shift toward real-time, data-driven corrections to support advanced applications for accurate quantification in research and clinical applications.

Despite these advancements, implementing effective head motion correction presents notable challenges, including the need for precise real-time tracking, which can be computationally intensive and susceptible to artifacts from rapid or complex head movements. For the systems described previously, integrating motion data with reconstruction algorithms should take into account the DOI effects, attenuation map mismatches and TOF precision, but signal degradation in low-count scenarios or interference from patient-specific factors (e.g., Parkinson) compromise quantification accuracy. Additionally, in hybrid PET/MR, aligning motion correction across modalities adds layers of complexity, requiring synergistic strategies.

### 3.2 Radiometabolites

Radiometabolite correction in brain PET applications is essential for accurate quantification, as radiolabelled metabolites can contaminate the IDIF derived from vascular regions, leading to overestimation of tracer uptake and biased kinetic parameters. Unlike invasive arterial sampling, which allows direct metabolite analysis, e.g., via radio high-performance liquid chromatography (radio-HPLC), IDIF relies on PET image data, where metabolites contribute to the total signal without differentiation, exacerbating issues in dynamic studies of neurotransmitters. The current status of the field reflects limited widespread adoption, with IDIF successfully implemented for only a minority of tracers (Table 1) due to challenges like PVE and inter-subject metabolic variability; however, recent advances include blood-free modeling approaches for early-phase tracer dynamics to estimate parameters with non-invasive kinetic modeling (Maccioni et al., 2024; Asch et al., 2025). Looking ahead, challenges persist in achieving reliable metabolite separation without blood samples for the majority of tracers.

**Table 1:** List of radiotracers with IDIF validation methods depicted by imaging systems, IDIF region estimation, technique for blood sampling and metabolite’s correction when applicable.

Radiotracer	Reference	System	IDIF Region	Metabolites	Technique
[^11^C]PIB	Lopresti BJ et al. (2005) J Nucl Med.	ECAT HR + PET	ICAs	Yes	AIF; HPLC
[^18^F]FLT	Backes H et al. (2009) J Nucl Med.	ECAT EXACT HR PET	ICAs	Yes	AIF; average metabolite correction
[^18^F]FDG	Zanotti-Fregonara P et al. (2009) J Cereb Blood Flow Metab.	ECAT HR+ PET	ICAs	No	AIF; blood calibration
[^18^F]FDG	Sundar LK et al. (2019) J Cereb Blood Flow Metab.	Siemens Biograph mMR PET/MRI	ICAs	No	AIF
[^15^O]H_2_O	Vestergaard MB et al. (2021) Neuroimage.	Siemens Biograph mMR PET/MRI	ICAs	No	AIF
[^18^F]GE-179	Galovic M et al. (2021) NeuroImage.	GE SIGNA PET/MRI	ICAs	Yes	5 venous samples; HPLC
[^18^F]FDG	Silvestri E et al. (2022) IEEE EMBC.	GE SIGNA PET/MRI	ICAs, superior sagittal sinus	No	No blood
[^18^F]MC225	Salvi de Souza G et al. (2025) Front. Nucl. Med.	uEXPLORER Total-Body PET	Aortic arch, ICAs	Yes	AIF; HPLC
[^18^F]FDG [^18^F]SynVesT-1 [^18^F]Flubatine [^11^C]LSN3172176 [^11^C]PHNO	Volpi T et al. (2025) J Nucl Med.	NeuroEXPLORER	ICAs, common carotid (CC)	Yes	AIF; HPLC

In a multi-tracer approach, overlapping signals and complex metabolite profiles amplify the challenges. Precise blood sample analysis is needed to separate the contributions of each tracer and their metabolites. However, traditional methods like radio-HPLC are cumbersome and time-intensive when dealing with multiple compounds, potentially introducing errors from incomplete separation or variable metabolism rates across tracers. In IDIF methods, these issues are exacerbated by the inability to distinguish tracer-specific metabolites directly from vascular regions, leading to biased quantification in brain regions and reduced reliability for dynamic studies. Future techniques may address these hurdles with advanced imaging reconstruction and signal processing, enabling separation of multi-tracer signals into the ICAs dynamic imaging. Chromatographic methods, such as radio-HPLC with automated detection (including tracer signal separation), could enable real-time separation of tracers and metabolites in a few (arterialized/venous) samples taken at later time points during multi-tracer PET, providing individualized IDIF with less manual processing and invasiveness. These approaches, combined with new brain-dedicated PET systems, could streamline multi-tracer protocols, enhance quantitative accuracy, and facilitate broader adoption in research.

## 4 Discussion

In summary, the evolution of brain PET scanners represents a transformative leap in simultaneous multimodal neuroimaging, offering unprecedented spatial resolution, high sensitivity and temporal precision that synergize with 7 T MR techniques. These advancements address longstanding limitations in PET quantification, particularly through enhanced IDIF methods that minimize invasive arterial sampling while improving accuracy in capturing the radiotracer bolus peak and reducing PVE in vascular structures.

From a broader perspective, IDIF in brain PET quantification is poised to become closer to a standard non-invasive tool as next-generation systems close gaps in resolution and sensitivity. By integrating AI-driven segmentation, advanced reconstruction algorithms with improved correction methods, and dynamic phantoms for validation, IDIF can evolve from a tracer-specific to a multi-tracer studies. This shift reduces patient burden and unlocks new possibilities for studying the brain, such as mapping multiple dynamic neurotransmitter systems, elucidating subtle pathologies in neurodegenerative disorders and tracking psychiatric conditions. In translational medicine, IDIF-enabled PET/MR could accelerate drug development by providing biomarker-driven endpoints for clinical trials, support personalized therapies for brain tumors or epilepsy, and enhance early diagnostics with multimodal data integration. However, realizing this potential requires overcoming residual challenges such as motion artifacts, radiometabolite corrections, and standardizations. Collaborative, interdisciplinary efforts are therefore needed to propel the field toward validated, blood-free, full-brain quantification.

## Data Availability

The data analyzed in this study is subject to the following licenses/restrictions: data protection rules. Requests to access these datasets should be directed to c.lerche@fz-juelich.de.
